# Impact of initial tumor volume on radiotherapy outcome in patients with T2 glottic cancer

**DOI:** 10.1007/s00066-014-0603-7

**Published:** 2014-03-04

**Authors:** T. Rutkowski

**Affiliations:** Department of Radiation Oncology, Maria Sklodowska-Curie Memorial Cancer Center and the Institute of Oncology, 44–101 ul. Wybrzeże Armii Krajowej 15, Gliwice, Poland

**Keywords:** TNM staging, Dose fractionation, Survival, Radiation oncologist, Intensity-modulated radiation therapy, TNM-Staging, Dosisfraktionierung, Überleben, Strahlenonkologe, Intensitätsmodulierte Strahlentherapie

## Abstract

**Background:**

The aim of this study was to quantify the impact of initial tumor volume (TV) on radiotherapy (RT) outcome in patients with T2 glottic cancer.

**Materials and methods:**

Initial TV was calculated for 115 consecutive patients with T2 glottic cancer who had been treated with definitive RT alone at a single institution.

**Results:**

The results showed strong correlations of TV with 3-year local tumor control (LTC) and disease-free survival (DFS). For TV ≤ 0.7 cm^3^, 3-year LTC was 83 %; for TV 0.7–3.6 cm^3^ this was 70 % and for TV 3.6–17 cm^3^ 44 %. Analysis of total dose vs. initial TV showed that larger T2 glottic tumors with a TV of around 5 cm^3^ (2–2.5 cm in diameter with 10^10^ cancer cells) need an extra 6.5 Gy to achieve similar 3-year LTC rates as for small tumors with a TV of 0.5 cm^3^ (~ 1 cm in diameter with 10^9^ cancer cells).

**Conclusion:**

Although classification of tumors according to TV cannot replace TNM staging in daily practice, it could represent a valuable numerical supplement for planning the optimal dose fractionation scheme for individual patients.

## Abstract (de)

**Hintergrund:**

Ziel der vorliegenden Studie ist quantitative Bestimmung des Einflusses des anfänglichen Tumorvolumens (TV) auf die Ergebnisse der Strahlentherapie (RT) bei Patienten mit Glottiskrebs im Krankheitsstadium T2.

**Material und Methoden:**

Das anfängliche TV wurde bei 115 nachfolgenden Patienten mit Glottiskrebs im Krankheitsstadium T2 gemessen, die ausschließlich mit RT in einer einzigen Einrichtung behandelt wurden.

**Ergebnisse:**

Die Ergebnisse ergaben eine starke Korrelation von TV mit dem Ergebnis der 3-jährigen lokalen Tumorkontrolle (LTC) und dem krankheitsfreien Überleben (DFS). Für TV ≤ 0,7 cm^3^ betrug das Ergebnis der 3-jährigen LTC 83 %, und entsprechend 70 % für TV 0,7–3,6 cm^3^ und 44 % für TV 3,6–17 cm^3^. Die Analyse der Gesamtdosis im Vergleich zum anfänglichen TV ergab, dass im Bereich des Glottiskrebs im Krankheitsstadium T2 größere Tumoren mit einem TV von etwa 5 cm^3^ (2–2,5 cm Durchmesser mit 10^10^ Tumorzellen) die Anwendung von zusätzlichen 6,5 Gy erfordern, um eine vergleichbare 3-jährige LTC wie bei kleinen Tumoren mit einem TV von 0,5 cm^3^ (~ 1 cm Durchmesser mit 10^9^ Tumorzellen) zu erreichen.

**Schlussfolgerung:**

Obwohl in der täglichen Praxis die Klassifizierung nach Tumorvolumen die TNM- Klassifizierung nicht ersetzen kann, könnte sie für einzelne Patienten eine wertvolle zahlenmäßige Ergänzung für die Planung der optimalen Fraktionierung der Strahlendosen darstellen.

For patients with T2 glottic cancer, conservative surgery, radiation therapy (RT) or a combination of both are considered standard therapy. The choice of treatment is based on the physician’s experience, hospital policy and patient willingness. In most centers however, RT is the initial treatment prescribed for T2 glottic lesions, with surgery reserved for salvage after RT failure. Although hemilaryngectomy or cordectomy produce comparable cure rates for selected T2 vocal cord lesions, RT is generally preferred: the major advantage of RT cover partial laryngectomy is better posttreatment voice quality [9–11].

The TNM disease stage is useful for surgeons, but not necessarily for radiation oncologists. The essential effect of radiation is cell killing. The degree of cell killing to be achieved depends on the initial number of cancer cells and dose fractionation planning should thus be tailored to this parameter. The T stage according to the Union for International Cancer Control (UICC) TNM classification system may not correlate with RT outcome if the same total dose is delivered to a given stage of cancer: different tumors of the same stage can differ significantly in terms of the initial number of cancer cells [1, 3]. It seems that for many tumors, particularly in the head and neck region, volumetric staging might be a more precise numerical parameter than TNM. At least for head and neck cancer, a number of authors have found a significant correlation between initial tumor volume (TV) and local tumor control (LTC) after RT [2, 4–7]. However, the majority of available data represent heterogeneous groups of patients, mostly in advanced stages of disease, with a relatively wide range of TVs. Among laryngeal tumors, the T2 stage of glottic cancer refers to infiltration of the larynx and impaired vocal cord mobility, irrespective of the wide spectrum of TVs. Therefore, the present study aims to analyze the impact of variation in initial TV on RT outcome in T2 glottic cancer.

## Materials and methods

### Patient characteristics

The study included 115 previously untreated consecutive patients with T2N0M0 glottic cancer who were treated with RT alone between 2002 and 2009. There were 104 men (91 %) and 11 women (9 %); age 40–80 years (mean 61 years). The clinical diagnosis of T2 glottic cancer was based on CT examination supplemented with information concerning tumor spread or superficial infiltration, as obtained upon endoscopic laryngeal examination. Neither positron-emission tomography (PET) nor MRI was routinely performed. Extension of the tumor into supraglottic regions was found in all cases. Impaired vocal cord mobility was not observed in isolation. In terms of histopathology, there were 24 (21 %) and 45 (39 %) well (G1) and poorly (G2, G3) differentiated tumors, respectively; in 46 patients (40 %), no data on this was available. Human papillomavirus (HPV) status was not examined in this group of patients.

In all cases, the primary TV was contoured on contrast-enhanced CT scans (3 mm slices). TV (cm^3^) was calculated using the volume algorithm of the Eclipse Treatment Planning System (Varian Medical Systems, Palo Alto, CA, USA). TV ranged from 0.16 to 17 cm^3^ (tumor diameter: 0.34–3.2 cm). Median hemoglobin concentrations before (Hb0) and after RT (Hb1) were 14.8 and 13.9 g/dl, respectively. An additional parameter representing the individual difference between Hb1 and Hb0, ΔHb, was also calculated. The median ΔHb was − 1.2 g/dl, (range − 4.6–1.1 g/dl). Hemoglobin concentration was not corrected in any way, neither before nor during RT.

### Treatment

All patients were treated using the three-dimensional intensity-modulated RT (3D IMRT) technique. The planning target volume (PTV) corresponded to treatment of the primary tumor with a minimum margin of 1 cm, to compensate for laryngeal motion. The high-dose clinical target volume (CTV) involved the majority of the larynx and involved nodes with a 1 cm margin. Remaining nodal levels were encompassed in the elective-dose CTV.

Total fractionated dose ranged from 45 to 74 Gy, given within an overall treatment time (OTT) ranging from 28 to 57 days. Because the dose per fraction ranged between 1.8 and 3 Gy, total dose was recalculated as the normalized total dose (NTD) if given in 2.0-Gy fractions (Gy_2_) using a linear-quadratic model (with an α/b value of 10.0 Gy).

### End points and statistics

The follow-up was at least 3 years. Cases lost from observation were censored. LTC was scored if there was no evidence of disease during at least 3 years. Local failure was defined either as persistent tumor (PT) at the completion of the RT or as local recurrence (LR) if disease reoccurred during follow-up after complete tumor clearance at the end of RT. Actuarial disease-free (DFS) and overall (OS) survival were calculated using the Kaplan–Meier statistic and the differences were assessed by the log-rank test. Raw data were used to analyze the relationship between NTD, TV and tumor cure probability (TCP). To evaluate the impact of TV and other clinical and treatment parameters (age, symptom duration, NTD, OTT, ΔHb) on treatment outcome (i.e. TCP), univariate analysis was first used. Variables which reached significance in this first step were then analyzed by multivariate Cox regression. A p-value of 0.05 was accepted as the level of significance.

## Results

Overall 3-year LTC was 71 %. There were 19 instances of PT (17 %) after RT and 14 cases of LR (12 %).

### Relationship between TV and LTC

**Fig. 1:**
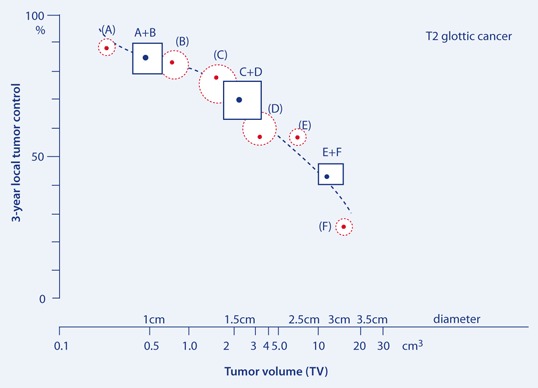
Measured 3-year local tumor control (LTC) of T2 glottic cancer as a function of tumor volume (*TV*). *Circles* are proportional to the number of cases, *squares* represent each of two-subgroups)

Data were subdivided into six groups (A–F), depending on TV. LTC rates and the incidence of failure in relation to initial TV are shown in Table 1. A strong and significant correlation of LTC with initial TV was observed. A sixfold increase in initial TV (on average from 0.5 to 3 cm^3^) translated into a decrease in LTC of about 30 % (from 89 to 57 %; Fig. 1). The significant correlation of TV with LTC was apparent when these were treated both as continuous and as categorized variables. A decrease in TV from about 17.1 to 0.5 cm^3^ leads to a threefold increase in LTC (from 25 to 84 %). Similarly, for tumors with TV > 1.6 cm^3^, the risk of local failure was almost four times higher than for the TV ≤ 0.5 cm^3^ (Table 2).

**Table 1:** Dependence of local tumor control (LTC) on initial tumor volume (TV) in T2 glottic cancer

TV group	No. cases	TV (cm^3^)	Diameter (cm)	Failure		LTC	3-year LTC
				PT	LR		
A	9	≤ 0.28	≤ 0.8	1	–	8	89 %
B	32	0.29–0.7	0.81–1.1	–	5	27	84 %
C	33	0.71–1.76	1.11–1.5	3	4	26	78 %
D	21	1.77–3.6	1.51–1.9	7	2	12	57 %
E	12	1.61–8.2	1.91–2.5	4	1	7	58 %
F	8	8.21–17.1	2.51–3.2	4	2	2	25 %
Intergroup comparisons							
TV groups			P-value				
(A + B) vs. (C + D)			0.05				
(C + D) vs. (E + F)			< 0.05				
(A + B) vs. (E + F)			< 0.005				

*PT* persistent tumor, *LR* local recurrence

**Table 2:** Local failure hazard ratio in relation to tumor volume

TV (cm^3^)	HR	95 % CI	*P*-value
≤ 0.5	1	–	–
> 0.5– ≤ 1	1.19	0.26–4.54	0.8
> 1– ≤ 1.6	1.77	0.39–8.09	0.4
> 1.6	3.93	1.06–14.54	0.03

*HR* hazard ratio, *CI* confidence intervals, *TV* tumor volume

Among the factors and parameters analyzed using the multivariate Cox model, ΔHb and TV were found to be significant independent risk features for local failure. An increase in ΔHb of 1 g/dl during the course of RT decreased the risk of local failure by 36 %. Independent of other factors, tumors with a TV larger than 1.6 cm^3^ had a significantly (threefold) higher risk of local failure than smaller tumors (Table 3).

**Table 3:** Results of multivariate analysis in terms of local failure hazard ratio

Variable	HR	95 %CI	*P*-value
Age	1.03	0.96–1.10	0.3
ΔHb	0.64	0.40–1.02	0.05
TV (> 1.6 cm^3^)	3.21	1.07–9.63	0.03

*HR* hazard ratio, *CI* confidence intervals, *ΔHb* difference in hemoglobin concentrations before and after radiotherapy,* TV* tumor volume

### Radiobiological rationale and the TV–NTD–LTC relationship

**Fig. 2:**
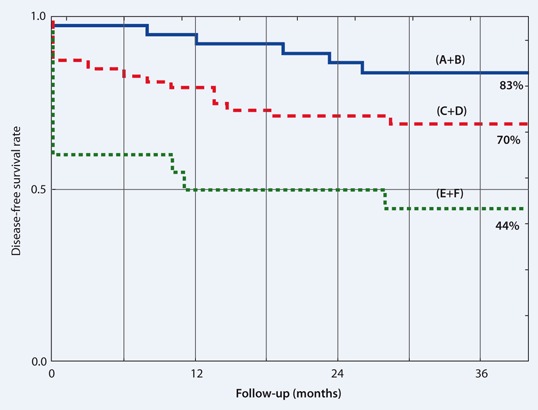
Actuarial disease-free survival (DFS) as a function of initial tumor volume (TV). Significant differences between groups A + B (*blue solid line*), C + D (*red dashed line*) and D + E (*green dotted line*)

**Fig. 3:**
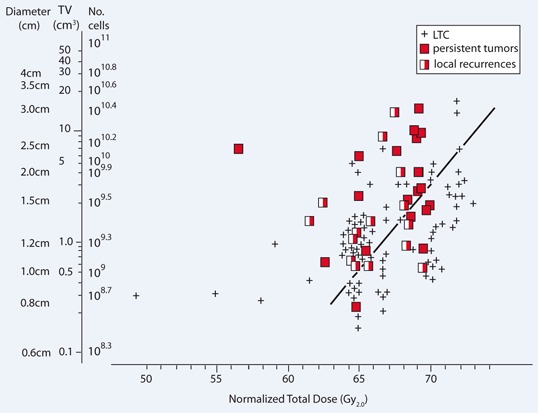
Theoretical iso-NTD_85_ curve (*black line*) according to tumor dimensions. *LTC* local tumor control

Actuarial DFS as a function of TV is illustrated by the Kaplan–Meier plot shown in Fig. 2. Raw data points for 3-year LTC, PT and LR are plotted within the coordinates of initial TV and NTD in Fig. 3. Except for 9 cases, NTD data points are distributed within a relatively narrow range of 65–73 Gy.

The effective D_0_ (_eff_D_0_) is the dose which reduces cell survival by one natural logarithm (e^−1^) for a particular fractionation regimen. For analysis of clinical data it is more useful to replace_ eff_D_0_ with D_10_, which is the dose that reduces survival by one common logarithm. An _eff_D_0_ of 2.8 Gy was selected (acceptable average value for small head and neck squamous cell tumors), which corresponds to a D_10_ of 6.5 Gy [7]. For tumors 0.5 cm in diameter (10^9^ cells), an NTD of 65 Gy_2.0_ translates into a TCP of about 85–90 %. If the number of tumor cells increases by one order of magnitude to 10^10^ (5 cm^3^), then a similarly effective NTD should be 71.5 Gy. Based on these assumptions, a theoretical iso-NTD_85_ (NTD required to give a 3-year LTC of 85 %) curve has been plotted (Fig. 3). At first glance, the iso-curve does not clearly separate control cases from failures because total doses were designed based on T stage, not on TV. However, it fits to the data set quite well. For small tumors (≤ 0.5 cm^3^) the NTD of 65 Gy_2.0_ gives a 3-year TCP of about 84 % (10/12) and for tumors with a tenfold larger volume (and with the cell number increased by one order of magnitude) the TCP is around 100 % if the NTD increases to 71.5 Gy_2.0_. Using the curve shown in Fig. 3, 3-year LTC rates were calculated in relation to a given NTD (Table 4). The NTD of 67 ± 2 Gy_2.0_ gave rise to a calculated 93 % 3-year LTC for very small tumors (tumor cell number ≤ 10^9^), which decreases significantly (*p* < 0.0001) to 33 % for 2–2.5 cm tumors and even further to 14 % for tumors larger than 3 cm. At an NTD of 72 ± 2 Gy_2.0_, the 3-year LTC was 100 % for both small and larger tumors.

**Table 4:** Calculated 3-year local tumor control (LTC) of T2N0M0 glottic cancer related to tumor volume (TV), i.e. no. cells, diameter and total NTD

No. tumor cells/diameter	3-year local tumor control	
	67 ± 2 Gy_2.0_	72 ± 2 Gy_2.0_
≤ 10^9^ ( ≤ 1 cm)	93 % (13/14)	100 % (3/3)
10^9.8^–10^10.1^ (2–2.5 cm)	33 % (5/15)	100 % (9/9)
10^10.1^–10^10.6^ (3–5 cm)	14 % (1/7)	100 % (3/3)

## Discussion

The fundamental goal of radical (not palliative) RT is to kill the last surviving cancer cell: if a single cell survives, local failure can likely be expected and the entire dose would have been “wasted”. Logically, the total dose and its fractionation should be tailored to the initial number of tumor cells, which more strongly correlates with TV than with tumor diameter or T stage. If tumor diameter doubles (e.g. 1–2 or 2–4 cm), TV increases eightfold and the cell number increases by about one order of magnitude. The design of dose fractionation schemes for individual patients depends on the clinical and, ever increasingly, on the molecular profile of the tumor, although TNM stage still plays major role. However, there is a relatively large variation in TV within a given T stage (N0M0) and it seems illogical to prescribe the same total dose to all tumors within a single T category.

The present study comprises a clinically homogenous group of 115 T2N0M0 glottic cancers and demonstrates a significant correlation between 3-year LTC and initial TV. An increase in TV from about 0.3 to 17 cm^3^ resulted in a decrease in 3-year LTC from 89 to 25 % (Fig. 1). Moreover, for patients with a TV of 1.6 cm^3^, the risk of local failure was four times higher than for those with a TV of 0.5 cm^3^. TV has been identified as a significant independent predictor for treatment outcome (Tables 2 and 3). This relationship also has a significant impact on 3-year DFS, which was significantly lower (about twofold) for larger tumors than for smaller ones. The prognostic impact of both TV and hemoglobin concentration on the outcome of patients with head and neck cancer was reported by Rudat et al. [12] in 68 patients with advanced tumors treated with chemoradiotherapy. The significant influence of Hb0 and Hb1 concentrations on RT outcome for patients with T2 glottic and epiglottic tumors had been also reported previously; furthermore, a significant negative correlation between Hb0, Hb1 and TV was noticed in this group of patients [13]. The correlation between TV and treatment outcome after RT has also been documented by other authors: Lo et al. [6] noted a significantly higher risk of local failure in T2 glottic cancer for TV greater than 4 cm^3^ (~ 2 cm in diameter). Kraas et al. [4]) chose their cutoff TV at 6 cm^3^ and measured the 2-year LTC for smaller tumors at 67 % compared with 43 % for larger ones (*p* = 0.07). For the same cutoff TV, Mancusco et al. [7] noted LTC rates of 83 and 46 % for smaller vs. larger tumors, respectively. A study by Hamilton et al. [2] focused on small glottic tumors showed 88 % vs. 31 % LTC rate for glottic tumors with TV smaller and larger than 1 cm^3^ (1.2 cm in diameter), respectively. However, the latter analysis consisted of only 50 cases. The results cited above correspond to those of the present study. The major difference is that other authors have used arbitrary TV cut off levels, whereas in the present study, the TV has been analyzed as a continuous variable. Using TV cutoffs we noted a 3-year LTC of 85 % for TV ≤ 0.7 cm^3^, 70 % for TV of 0.7–3.6 cm^3^ and 45 % for TV of 3.6–17 cm^3^.

All these studies require very careful interpretation, since the dose fractionation for individual patients was likely designed mainly on the basis of T category, with TV not being considered as an important pretreatment determinant (at least not in the way presented here).

On the other hand, for relatively small T2N0M0 glottic tumors it is hard to believe that hypoxia and repopulation may have important impact on the response to a given NTD. If the NTD is more or less constant, the difference in the initial number of tumor cells (various TV) may be a predominant factor. The wide spread of individual NTD data points within each of the TV compartments is shown in Fig. 3. This demonstrates that total doses were not designed according to initial TV, but were likely intuitively larger for small but infiltrating tumors. For an NTD of 65 Gy_2.0_, failures were observed for tumors with TV > 4 cm^3^; whereas for 67 Gy_2.0_, all tumors with TV ≤ 8 cm^3^ were locally controlled. Despite some uncertainties, the volume–dose–response relationship fits radiobiological rationale. The theoretical iso-curve in Fig. 3 fits to the analyzed data set quite well. An NTD of 67 ± 2 Gy_2.0_ produced a 3-year LTC of 93 % for small T2 glottic cancer with a TV of 0.5 cm^3^ (1 cm in diameter) or less. For larger tumors with a TV of ≥ 5 cm^3^ (≥ 10^9.8^ cells), the 3-year LTC was significantly (*p* < 0.0001) lower at 33 %. Delivery of an NTD of 72 ± 2 Gy_2.0_ gave rise to 100 % 3-year LTC, independent of tumor volume. Therefore it seems reasonable to postulate that for T2N0M0 glottic cancer, a tenfold increase in TV (increase in the number of tumor cells of about one order of magnitude) needs an extra dose of about 6.5 Gy to achieve similarly high 3-year LTC.

Volumetric staging quantified by measurement and calculation of TV will not replace TNM staging, but it can be a valuable numerical supplement for designing optimal dose fractionation planning for individual patients.

## Conclusion

Despite some limitations, the present study clearly documents the significant impact of initial TV on the RT response of T2N0M0 glottic cancers. Although the size of the total dose was not designed in relation to TV, a significant volume–dose–response relationship was noted. The TV measurements do not replace TNM categories, but could provide a valuable numerical supplement for the design of optimal dose fractionation schemes for individual patients.

## Compliance with ethical guidelines

### Conflict of interest.

T. Rutkowski states that there are no conflicts of interest.

## Appendix

Poissone statistics correlate tumor control probability (TCP) with cell survival rate as follows:

1 $ {\rm{TCP = }}{{\rm{e}}^{ - x}} = {{\rm{e}}^{ - {\rm{(IC}} \cdot {\rm{SF)}}}}, $

Where x is the average number of surviving cancer cells per tumor, which results from multiplication of initial cell number (IC) by the surviving fraction (SF).

Simple relationship between SF and a given total dose formula:

2 $ {\rm{SF = }}{{\rm{e}}^{ - {\rm{TD/effD0}}}}, $

Where _eff_D_0_ reduces survival rate to e^−1^

For analysis of clinical data is more useful to replace _eff_D_0_ by D_10_, which reduces SF by one order of magnitude and it equals:

3 $ {{\rm{D}}_{{\rm{10}}}} = 2.3{ \cdot _{{\rm{eff}}}}{{\rm{D}}_{\rm{0}}}, $

Example: if IC = 10^9^ cancer cells (0.5 cm^3^ tumor) than TD = 10 x D_10_ is needed to achieve x = 0.1, which gives TCP = 90 %.
